# Evaluation of Frequency of Four Common Nasal Anatomical Deformities in Primary Rhinoplasty in A Tehran Plastic Surgery Center

**Published:** 2014-07

**Authors:** Mehdi Eskandarlou, Sadrollahe Motamed

**Affiliations:** 1 Department of Surgery, Hamadan University of Medical Sciences, Hamadan, Iran;; 2 Department of Plastic Surgery, Shahid Beheshti University of Medical Sciences, Tehran, Iran

**Keywords:** Alar cartilage, Radix, Middle vault, Tip projection

## Abstract

**BACKGROUND:**

In rhinoplasty, functional and cosmetic problems including imbalance between the nasal subunits and face are aimed to be corrected. So there is a need for careful preoperative evaluation and treatment of these patients. According to functional and aesthetic effects of these variables in rhinoplasty, evaluation of the frequency with focus to diagnostic methods was undertaken.

**METHODS:**

In a descriptive study, 100 volunteer patients for primary rhinoplasty were enrolled. After history taking, nasal examination, desirable paraclinical work up and photography taking, presence of 4 anatomical variants was evaluated on the base of definition about normal and abnormal characteristic of organ.

**RESULTS:**

Twenty nine male and 71 female patients underwent primary rhinoplasty. Open rhinoplasty was done in 85 and the close technique in 15 patients. 77% of patients had at least one of four anatomical nasal variations. The most common anomaly was alar cartilage malposition (51%) and frequency of others was low radix (36%), inadequate tip projection (35%) and middle vault collapse (15%). Frequency of low radix in male patients was 2.5 times more than females.

**CONCLUSON:**

Success in rhinoplasty needs careful nasal analysis and evaluation. As at least one of four anatomical nasal variations is diagnosed before surgery, the correction has an important role on the outcome. As frequency of middle vault narrowing was low, a definitive diagnosis of alar cartilage malpositioning seems necessary in surgical exploration. Needs for correction and methods of treatment of variants can be based on dynamic interplay between nasal subunits.

## INTRODUCTION

Rhinoplasty is a surgical intervention on the nose for shaping, contouring and adequate balancing between nose and other aspect of the face. It is one of the most common aesthetic surgeries in Iran, used to correct many nasal problems and deformities on the radix, dorsum, septum, tip and so on.^1^ In primary rhinoplasty, patients have a good chance to receive a successful aesthetic and functional outcome. Accordingly, this needs careful nasal analysis and surgical planning accompanied with using a suitable surgical technique.^2^^,^^3^ Among many anatomic deformities that are candidate for patients undergoing rhinoplasty, four variants are more important and common, so if surgeons do not focus on them, unpleasant results would be expected postoperatively.^1^^,^^2^ These are alar cartilage malposition, inadequate tip projection, middle vault collapse and low radix. 

Localization of radix can be undertaken with several methods (Figure l). Radix defines the most concavity part of cephalic dorsum.^5^ Normal distance of radix to inner canthus is 6 mm and distance between corneal plane and radix plane is about 9-14 mm.^5^ Ideal radix projection (height) is 0.28 of ideal nasal length.^5^

**Fig. 1:**
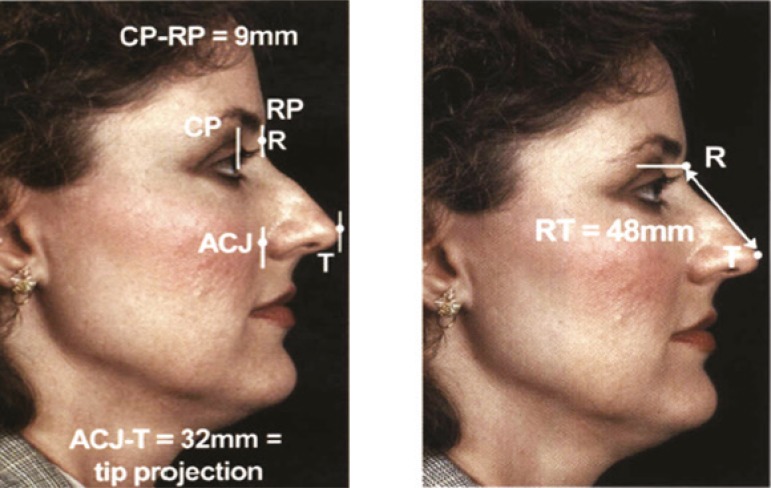
Anatomic localization of the radix. H. Steve Byrd, James D. Buri. Dimensional approach to Rhinoplsty: Perfecting the aesthetic balamce between the nose and chin. Dallas , Volume 1, Chapter 7. 2002: 120-121.

Half or two third of cephalic nasal vault is bony structure and two third of caudal nasal vault is constituted by upper and lower lateral cartilage.^2^^,^^3^^,^^6^ A portion of anterior aspect of upper lateral cartilage transversely curves and attaches to septal cartilage and performs middle nasal vault.^5^^-^^7^

**Fig. 2:**
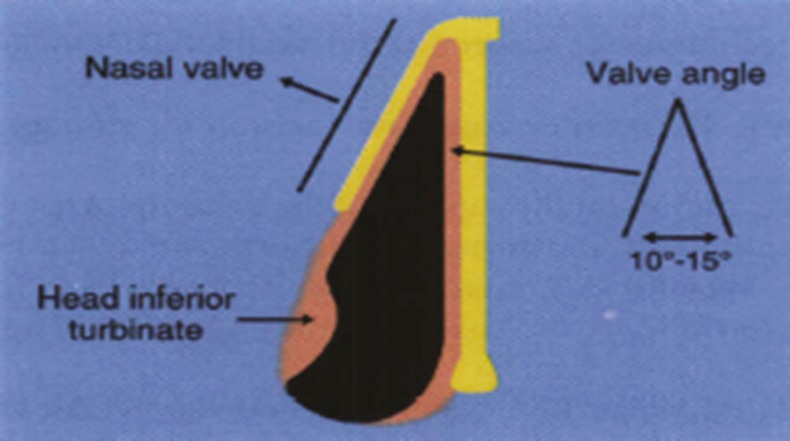
Angle of internal nasal valve and determination of middle nasal vault respect to proximal and distal nasal vault. Rohrich RJ, Adams Jr WP, Gunter KP. Advanced rhinoplasty anatomy. Dallas, 2002;1:17.

Angle between septal and upper lateral cartilage is 10-15 degrees (Figure 2). Preserving of this relationship is important for decreasing of air flow resistance in middle vault as well as formation of an even dorsal aesthetic line. Middle vault narrowing or collapse defines narrowing of the middle vault to less than 75% of proximal (cephalic) or distal (caudal) third of the nose.^2^^,^^5^

Nasal tip or apex of lobule demonstrates the amount of nasal prominency respect to midface. This projection depends on the size and quality of upper lateral cartilage, alar dome cartilage, medial crura, support of dome by septum and thickness of coverage.^2^^,^^5^ Adequacy of nasal tip projection defines when 50-60 percent of the nasal tip lies anterior to vertically crossed lip line.^8^^,^^9^ Alternatively, distance between alar facial groove to nasal tip is about 0.67 ideal nasal length. Ideal nasal length is 0.67 of midface.^2^^,^^8^ Normally, tip lobule places over the anterior septal angle.^2^

**Fig. 3:**
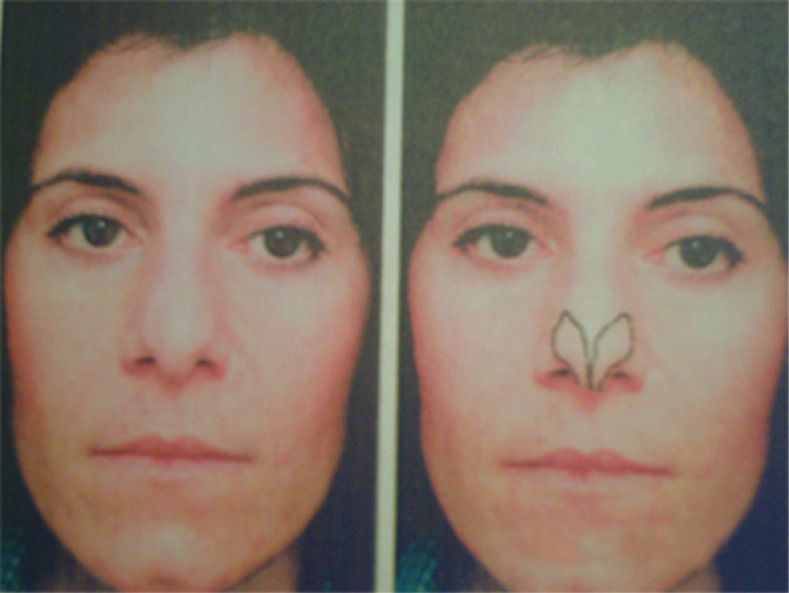
Determination of lower lateral cartilage axis respect to inner and outer canthus on the surface anatomic of nose. Constantian MB. Common problems in anatomy and proportion. Constantian Rhinoplasty. Chapetr 15, 2009;2:917

Normally medial third of lower lateral cartilage is parallel to alar rim and then through genua has cephalade rotation with an angle about 30-40 degrees, so that long axis of lateral crura is directed to lateral canthus.^2^^,^^10^^,^^11^ In Figure 3 it is shown that if the angle increase more than 45 degrees, the long axis of lateral crura is directed to medial canthus and represents lateral crura malpositioning.^6^^,^^11^^,^^12^ In severe form of alar cartilage malpositioning, lateral crura would be parallel to dorsal septum and present as round, boxy and parenthesis deformity of nasal tip. With fine preoperative nasal analysis, surgeons would be able to detect and address the problems that patient has not any concern about them. With attention to these obscure problems in primary rhinoplasty, we would have a perfect aesthetic and functional nose post operatively and in spite of this secondary rhinoplasty would be inevitable.^1^^,^^2^^,^^4^ In this study, evaluation of frequency of four common nasal anatomical deformities in primary rhinoplasty in a tehran plastic surgery center was undertaken. 

## MATERIALS AND METHODS

A descriptive study with cooperation of 100 volunteer patients was done for primary rhinoplasty in a Tehran university hospital. In order to have a careful diagnosis, we used three methods for evaluation of nasal deformity or anatomic variants including (i) Analysis of nasal deformities according to preoperative clinical examination, (ii) Analysis of nasal deformities according to frontal, profile and basal view photography with reproductive ratio 1:1 (life size) and (iii) Intra-operative findings. 

Patients with history of trauma to nose and congenital anomaly of face and nose were excluded from the study. With special attention to patient’s request and desire for rhinoplasty, firstly internal nasal examination was undertaken to evaluate internal valve, external valve, septum and turbinates. Then, four anatomic variants were evaluated either with direct examination of nose and its proportion to the face or in photography. All steps for a classic rhinoplasty including para-clinical evaluation, diagnosis and treatment were considered for studied patients without any other intervention. 

In profile view, the deepest portion of the nasal dorsum was marked. The vertical line that was drawn through this point was called radix plane. In profile photography, the most prominent point of cornea was marked, while patients had straight forward gaze. Through this point, vertical line was drawn so called corneal plane (Figure 1). Normally, the distance between these two planes was considered 0.28 of ideal nasal length or equal to 9-14 mm.^5^ More than this amount was called low radix. A line was drawn from alar facial groove to tip defining point. If the length of this line was less than 0.67 facial length it is called inadequate tip projection.^1^^,^^6^ Ideal nasal length was the distance of radix to tip defining point and equal to 0.67 midface.^2^^,^^4^^,^^7^

Alar cartilage position was evaluated by clinical examination, photography and intra­operative findings. Various factors could affect this variant such as quality of alar cartilage and thickness of nasal coverage. Accordingly, in this study; the most definitive method for determination of alar cartilage position was considered to be operative exploration. In frontal view of photography and during clinical examination if it is possible, the long axis of lateral crural cartilages was drawn. Direction of the axis respect to canthi and the angle of it to alar rim were evaluated (Figure 3). 

Intraoperatively, lateral crura was explored. Long axis of the cartilage was marked by methylene blue. Anterior (medial) third of the cartilage was parallel to alar rim and then with angle of 30-45 degrees is directed to lateral canthus. Angle of cephalic divergence more than 45 degrees led to medial canthus deviation of lateral crura, called alar cartilage malpositioning. 

Dorsal aesthetic line was drawn over the nasal dorsum either in patient or in his (her) photography and the distance of the lines were measured at the level of middle vault (Figure 2). The size of middle vault less than 75 percent of width of proximal or distal third of nasal dorsum was considered middle vault narrowing.^2^^,^^5^

Alternatively, presence of inverted V deformity in frontal view of photography or nasal examination could be defined middle vault narrowing.^2^ Also collapse of lateral nasal wall with resting or forceful inspiration could represent the problem.^1^^,^^2^ Presence of middorsal notch in profile view of photography or clinical examination revealed middle vault collapse «too». 

In frontal view by clinical examination and photography, we could evaluate lateral crural malpositioning and middle vault collapse. These two findings disturbed nasal function and aesthetic. In profile view, tip projection and radix position were evaluated and the variants influenced the nasal aesthetic. 

## RESULTS

In this study, 100 patients including 29 males and 71 females with age range of 18-42 years (mean age of 24 years old) who underwent primary rhinoplasty were enrolled. In 15 cases (7 males and 8 females), rhinoplasty technique was a closed and in 85 cases (22 males and 63 females) was an opened technique. Twenty three percent of rhinoplasty patients did not have any one of four common anatomic variants but 77 percent of patients had at least one of the four anatomic variants. 

Forty six patients had at least one of the four anatomic variants. Forty six patients had one variant, 23 patients had two variants, 5 patients had three and three patients had all four anatomic variants. Fifty one patients (15 males and 36 female) had alar cartilage malposition. In 44 patients, diagnosis of alar cartilage malposition was on the base of preoperative clinical examination. Operative exploration of lower lateral cartilage in all of these 44 patients also revealed alar cartilage malpositioning (Table 1). 

**Table 1:** Comparison of preoperative and intraoperative diagnosis of alar cartilage malposition in 100 primary rhinoplasty patients.

Sex	Diagnosis of alar cartilage malposition by clinical examination No. (%)	Diagnosis of alar cartilage malposition by operative exploration No. (%)
Male	14 (48.5)	15 (51.5)
Female	30 (49.5)	36 (50.5)
Total	44 (45.8)	51 (54.2)

Two of our studied patients had alar cartilage malposition in clinical examination but intraoperative finding did not emphasize this deformity. In our study, 35 percent of patients (12 males and 23 females) had inadequate tip projection. The results of radix position evaluation were shown in Table 2. Fifteen patients had low radix and 21 patients (11 males and 10 females) had caudal positioned radix. 

**Table 2:** Frequency distribution of radix position in 100 primary rhinoplasty patients according to sex.

**Parameter**	**Male No. (%)**	**Female No. (%)**
Lower or caudal position radix	18 (62.5)	18 (25)
High radix	7 (24.2)	25 (35.2)

Evaluation of middle vault narrowing was shown in Table 3. Fifteen percent of our studied patients had middle vault collapse. 

**Table 3:** Frequency of middle vault narrowing in 100 primary rhinoplasty patients according to sex

**Parameter**	**Male No. (%)**	**Female No. (%)**
Narrow middle vault	5 (17)	10 (14.5)
Normal middle vault	24 (83)	61 (85.5)

## DISCUSSION

Success in primary rhinoplasty is dependent to attention to patient’s desire and her (his) incentive for rhinoplasty, evaluation of nasal facial component relationship, evaluation of nasal subunits to total nose relationship, diagnosis of anatomic variants, careful surgical planning and employment of meticulous surgical technique. Correction of the real deformity that is explained by patient has important role for satisfaction of patient and surgeon postoperatively. But surgeons must preoperatively demonstrate and address occult nasal variants or deformities in order to get a good aesthetic and functional result. 

Review of literatures show that between several nasal deformities that indicate rhinoplasty, there are four fundamental and basic anatomic variants which their diagnosis or avoiding to their development during primary rhinoplasty would lead to a satisfactory aesthetic and functional outcome.^1^^,^^2^ This subject is emphasized by observing a high incidence (77%) of 4 common anatomic variants in our studied patients. Comparison of this study to another one has been shown in Table 4.^1^

**Table 4:** Comparison of the frequency of four anatomic variants in rhinoplasty in the present study and Constantian studies.^1^

**Study**	**Low radix (%)**	**Inadequate tip Projection (%) **	**Alar cartilage malposition (%)**	**Middle vault** **collapse (%) **
Constantian study	50	30-40	50	40
Our study	36	35	51	15

Frequency of middle vault narrowing and low radix in our study were significantly low. Radix positioning can impact nasal subunits proportions and overall aesthetic of the nose. Radix position is evaluated in cephalic-caudal and anterior-posterior axis.^1^^,^^2^^,^^5^

Normal positioning of the radix in cephalic-caudal axis is between the level of upper lid margin and supratarsal fold with eyes in straight forward gaze. The distance between corneal and radix plane in anterior-posterior axis must be 0.28 of ideal nasal length.^5^ Lower or higher positioning of radix in both axis not only disturb aesthetic of the nose but also affect our concept about another subunits of the nose specially nasal base and length.^1^^,^^2^^,^^5^

Low positioning radix results short appearance of nasal length and increase of tip projection and nasal base seems to be more than its real size.^1^^,^^2^ Frequency of low radix in our male patients was 2.5 time more than female. On the other hand, frequency of low radix in all 100 studied patients was less than Constantian series (Table 4). 

Asian nose typically presentes with low dorsum, low radix, thick coverage, dorsal hump with more caudal positioning and higher incidence of alar cartilage malpositioning.2 In our study, the incidence of low radix was less than frequency of Asian race that would be expected. One reason of this difference may be due to less number of our male patients. On the other way, we had a high incidence of radix position respect to another studies. High radix positioning in female patients of our study was more. Among 15 out of 36 patients with low radix, it was necessary to correct the deformity with crushed cartilage to get a suitable balance. 

Inadequate tip projection in our study was relatively equal to another study (Table 4). In these patients, tip lobule was not placed over anterior septal angle. When this variant had not been noticed preoperatively, surgeons try to resect the dorsum in order to improve the tip projection. This procedure results supratip deformity and insufficiency of internal and external valve. In 27 out of 35 patients with inadequate tip projection deformity was corrected with columellar strut and tip sutures but in another 8 patients, we needed to add onlay tip «too». Modalities of treatment of this variant are medial crural sutures, clumellar strut, tip onlay grafts or altogether according to need for high projecting of tip.^13^^,^^14^

The least common variant in this study was middle vault narrowing. (Table 3). Middle vault collapse affects both nasal aesthetic and function. Middle vault would be three kinds including (i) Normal middle vault: In this form the distance between dorsal aesthetic lines is 6-8 mm in female and 8-10 mm in male,(ii) Narrowed middle vault: In this type the angle between dorsal septum with upper lateral cartilage is less than 10-15 degrees. This cause disturbance of nasal function and may be present as dorsal nasal depression, and (iii) Wide middle vault: Presence of this variant causes wide dorsum. Trying to correct this deformity can led to relatively middle vault narrowing and nasal dysfunction.^5^

Review of literature reveal that middle vault collapse and internal valve narrowing in primary rhinoplasty was about 4 times and in secondary rhinoplasty, 12 times more common in respect to nasal obstruction due to septal deviation.^1^^,^^2^ Predisposing factors for development of primary rhinoplasty are big dorsal hump, short nasal base and low dorsum.^2^^,^^5^

In our study, frequency of middle vault collapse was less than other studies. In 11 out of 15 patients, we corrected this variant with unilateral and in 4 another patients with bilateral spreader graft. Treatment of middle vault collapse can be dorsal onlay graft or unilateral or bilateral spreader graft and these modality dependent to functional or aesthetic impact of middle vault collapse over the nasal dorsum and side wall.^13^^,^^14^ The most common anatomic variant in our study was alar cartilage malpositioning and its incidence and male to female ratio did not have any significant statistical analysis difference (Table 3 and 4). This deformity firstly was described by Sheen and its importance was affliction of nose aesthetic, hazard of lower lateral cartilage transection during intracartilaginous incision and inability to support of external valve.^1^^,^^2^^,^^5^ Many authors believe that the diagnosis of alar cartilage malpositioning and adequacy of tip projection were determined preoperatively.^1^^-^^3^ But in our study, we had 7 from 100 patients without any signs of alar cartilage malposition preoperatively and deformity was diagnosed with surgical exploration. The causes of inability to preoperative diagnosis of this variant were thickening of the nasal coverage and paucity of fibrofatty tissue at caudal nasi. Therefore, in spite of other authors’ idea, it seems that the best diagnostic procedure of alar cartilage malpositioning is operative exploration and the mere diagnosis on the base of clinical examination and photography is not trustful. In 37 out of 51 patients with alar cartilage, malpositioning correction of deformity was done only by lateral crural strut graft but in 14 patients, we had to place strut graft accompanied with repositioning of lateral end of alar cartilage in order to get a desirable nasal aesthetic and balance. Treatmnet of alar cartilage malpositioning may be resection and relocation of the lateral crura, (to support the external valve) or cartilage grafts to lateral crura.^2^^,^^13^^,^^15^^,^^16^

This study showed that 77% of primary rhinoplasty patients had at least one of 4 common variants. The most common variant was alar cartilage malposition (50%) followed by inadequate tip projection. Both of these variants had similar frequency with other studies. Diagnosis of alar cartilage malposition is not always possible preoperatively and definitive diagnosis is made by surgical exploration. 

Lower incidence of low radix positioning in our study was unexpectedly against to Asian nose. Frequency of low radix in our male cases was 2.5 times more than female patients while frequency of high radix in female patients was more than male. For better conclusion about radix positioning in Iran, more cases of study are recommended. The least frequency of variant in our study was middle vault collapse that had equal incidence in both sexes. Success in rhinoplasty needs careful nasal analysis and evaluation and as at least one of four anatomical nasal variations was diagnosed preoperatively or it is predicted to their occurrence, their correction may be necessary (but not always) with respect to dynamic interplay between nasal zones. As frequency of middle vault narrowing was low, a definitive diagnosis of alar cartilage malpositioning seems necessary in surgical exploration. 

## CONFLICT OF INTEREST

The authors declare no conflict of interest. 

## Footenote

Please cite this paper as:

Eskandarlou M, Motamed S. Evaluation of Frequency of Four Common Nasal Anatomical Deformities in Primary Rhinoplasty in A Tehran Plastic Surgery Center. World J Plast Surg 2014;3(2):122-128.
